# Individual, family, and environmental determinants of vision-related quality of life of children and young people with visual impairment

**DOI:** 10.1371/journal.pone.0294532

**Published:** 2023-11-16

**Authors:** Ana Šemrov, Valerija Tadić, Mario Cortina-Borja, Jugnoo Sangeeta Rahi, on behalf of The Resilience and Childhood Visual Impairment Study Group

**Affiliations:** 1 Population, Policy and Practice Research and Teaching Department, Great Ormond Street Institute of Child Health, University College London, London, United Kingdom; 2 Ulverscroft Vision Research Group, University College London, London, United Kingdom; 3 School of Human Sciences and Institute of Lifecourse Development, University of Greenwich, London, United Kingdom; 4 Institute of Ophthalmology, University College London, London, United Kingdom; 5 Great Ormond Street Hospital NHS Foundation, London, United Kingdom; University of Ulster, UNITED KINGDOM

## Abstract

Childhood visual impairment can have a significant impact on an individual’s development. To improve clinical care and develop appropriate psychosocial interventions of these patients, it is necessary to understand the contributing and modifiable factors that both identify individuals in greater need and could be targeted in interventions. Here we investigate the broader individual, family, and environmental factors associated with vision-related quality of life (VQoL) of children and young people with visual impairment (CYP-VI). Data for this cross-sectional study were collected from September 2014 to May 2017 to develop and validate two vision-specific patient-reported outcome measures (PROMs) for CYP-VI. Patients were recruited from 22 hospitals in the United Kingdom and were aged 7–18 years with visual impairment as per WHO criteria. Participants self-completed the two PROMs, VQoL and Functional Vision Questionnaires. Clinical characteristics were extracted from medical records. Their carers provided information on family sociodemographic backgrounds. Associations between the VQoL scores and other factors were examined using Spearman’s correlation, Kruskal-Wallis, Wilcoxon rank-sum tests, and quantile regression models. The sample consisted of 152 CYP-VI (67 females). Better VQoL was significantly associated with better functional vision overall (*r*_Spearman_ = –0.52), parent-reported absence of additional chronic conditions (*d*_Cohen_ = 0.46), attending mainstream (versus other) school (*d*_Cohen_ = 0.44), higher socio-economic status (*r*_Spearman_ = 0.17) and higher parental education level (*r*_Spearman_ = 0.20). No other investigated factors were significantly associated with VQoL. The final quantile regression model included functional vision scores and the presence of additional health condition. Variation in self-reported VQoL in CYP-VI can be partly accounted for by factors relating to the clinical status of the affected child and, more importantly, by non-health-related factors. This needs to be considered in clinical practice when assessing vision-specific outcomes and providing support to CYP-VI, as well as in the development of future interventions.

## Introduction

Vision has a vital role in early human development [1–3]. Thus, it is not surprising that visual impairment or blindness (**VI**) in childhood, while uncommon [4], presents several developmental risks and can directly and cumulatively affect an individual’s development [5, 6]. However, the consequences of childhood VI go far beyond the developmental delay and challenges related to daily living [7]. Some children and young people with VI (**CYP-VI**) may be at risk of impaired quality of life (**QoL**) [7, 8], poor psychosocial adjustment [9], low self-esteem [10], and poor social and educational outcomes [11–17]. CYP-VI can experience difficulties fitting in and making friends and are frequently teased and bullied by their peers [6, 7, 13, 18–21].

The paediatric literature on other chronic health conditions indicates that individuals with severe disabilities or conditions can report good psychosocial QoL regardless of their impairments [22–27]. It also shows QoL is associated with different sociodemographic, clinical, child and family characteristics [26–33]. Therefore, it is important to identify and understand the specific factors that shape QoL of CYP-VI to prevent avoidable adverse outcomes and to maximise the positive QoL outcomes in individuals at risk [9, 10, 27, 34, 35]. However, there is very limited literature on factors that may influence QoL of CYP-VI specifically [36–38].

Patient-Reported Outcome Measures (**PROMs**) are increasingly used to evaluate holistically the impact of a health condition on the patient’s life from their own perspective [39–44]. Two recent complementary PROMs developed specifically for CYP-VI assess their vision-related quality of life (**VQoL**) [39, 40] and functional vision (**FV**) [41, 42]. They provide an age- and stage-appropriate method to assess the impact of VI on the child’s life experience and, thus, the outcome measures to delineate the factors that shape VQoL in visually impaired children and young people.

We used data collected from a representative sample of CYP-VI who completed these age-specific vision PROMs [40, 42] during the final stage of instrument development to investigate which broader sociodemographic, family, clinical, and environmental characteristics are related to self-reported VQoL. Our aim was to identify potential modifiable factors that could be targets for intervention to improve VQoL in CYP-VI.

## Methods

### Study design and setting

Data were collected between September 2014 and May 2017 as part of a larger programme of research developing and validating age- and stage-appropriate vision-specific PROMs for CYP-VI aged 8 to 18 years, specifically for the final phase of validation through a large-scale cross-sectional postal survey [40, 42].

The study was approved by the National Health Service Research Ethics Committee for Essex and East of England, United Kingdom (REC 12/EE/0455) and followed the tenets of the Declaration of Helsinki. The analysis presented here was part of the original ethics approval. All participants gave informed written consent if ≥16 years or informed written assent if <16 years alongside informed written parental consent. Participation in this study was voluntary.

### Participants

Patient identification and recruitment for this study have been reported in detail previously [40, 42]. Participants were visually impaired children and young people with visual acuity in the better eye of 0.48 Logarithm of the Minimum Angle of Resolution (**LogMAR**) or worse (worse than 6/18 Snellen), or additional visual defects causing VI as a result of any visual disorder. Those with other significant impairment that would prevent them from self-reporting and, therefore, self-completing the PROMs were not eligible. The achieved sample of 152 CYP-VI was representative of the overall target group in the United Kingdom (**UK**) with respect to sociodemographic and clinical characteristics [40]. All were attending an Ophthalmology Department at one of 22 hospitals across the UK from which the study’s sample was recruited.

### Measurements

CYP-VI self-completed the two age-appropriate PROMs (the Vision-Related Quality of Life Questionnaire for Children and Young People, **VQoL_CYP** [40]; and the Functional Vision Questionnaire for Children and Young People, **FVQ_CYP**) [42]. The VQoL_CYP captures the child’s perception of the impact of VI in terms of social relationships, emotional well-being, independence, and autonomy. The VQoL_CYP version for children aged 8–12 has 20 items, and for young people aged 13–18 years contains 22 items. The FVQ_CYP captures self-reported difficulty completing everyday activities requiring vision. The FVQ_CYP version for children aged 8–12 has 28 items, and the one for those aged 13–18 contains 38 items.

Both PROMs have been validated and calibrated using Rasch measurement theory [45] with the ability to transform the scores to a scale 0–100, treated as continuous, ratio-level data, and compared, despite variation in the number and wording of items. A higher score on the VQoL_CYP demonstrates better VQoL, while a higher score on the FVQ_CYP indicates worse functional vision (i.e., greater difficulty).

Participants’ age, gender, and clinical characteristics were retrieved from the hospital electronic records and used with their (or parental) consent. The child’s parent or main carer reported on the family and other sociodemographic characteristics using a structured family background questionnaire developed for this study. Detailed information on how these data were coded for the analysis can be found in S1 File.

### Procedures

A study pack with invitation letters, the age-appropriate child/young person and parent information sheets, consent and assent forms, and the age-appropriate instruments version in large print were sent to the participants by post. Completed consent/assent forms and the questionnaires were returned to the research group using a postage-paid envelope also provided within the study pack.

### Data analysis

Analysis was performed in R (version 4.1.2 GUI 1.77) [46] on Rstudio (version 2022.02.3.492) [47], and with IBM SPSS 27.0 [48]. An overall VQoL score was the outcome measure. Participants that did not respond to ≥20% of the items on VQoL_CYP or FV_CYP were excluded from the analysis (as per guidelines). For cases with <20% missing observations, data were imputed as the average score given by the participant on other items in the VQoL_CYP. Overall scores for CYP-VI were calculated and transformed to Rasch-scaled scores published in the instrument’s scoring instructions [40, 42].

Clinical factors considered in the analysis were severity of VI, timing of onset of vision loss, rate of deterioration of vision, and presence of additional chronic conditions or impairments affecting the child’s development.

Sex, age, and ethnicity of the child were considered alongside family structure, carer’s age, ethnicity, education, employment status and occupational level, number of siblings, birth order of the participating child, carers’ and siblings’ VI and health status. School type was considered as a broader environmental factor.

Socio-economic status was analysed using housing tenure, number of cars owned, and the index of multiple deprivation (**IMD**) based on the UK postal code [49] as the standard rank-level metric of relative deprivation.

Descriptive statistics of self-reported PROMs scores were calculated and stratified by measured associated factors. The assumption of normality for continuous variables was assessed using D’Agostino’s *K*2 test [50], *z*-tests of skewness and kurtosis, and screening of histograms [51]. Following this, Spearman’s correlation coefficient, Kruskal-Wallis test, and Wilcoxon rank-sum test were used to examine the relationship between the CYP-VI self-reported outcome measure and other factors.

Because the outcome variable was non-normally distributed, quantile regression models [52] for medians were fitted to estimate the effect of associated factors on the outcome measure. Only variables that showed a significant relationship with VQoL scores were used in quantile regression analysis.

## Results

Of 152 participating CYP-VI, two were excluded from the analysis due to missing data above the pre-specified threshold (one each for the VQoL_CYP and the FVQ_CYP instruments).

Normality test using *z-*scores for skewness and kurtosis was used to assess the distribution of VQoL_CYP and FV_CYP scores. D’Agostino’s *K*^2^ test showed both VQoL_C (*p <* .001; *z*_skewness_ = 3.18; *z*_kurtosis_ = 4.37) and FV_CYP (*p <* .001; *z*_skewness_ = –0.90; *z*_kurtosis_ = 4.57) scores were not normally distributed.

The self-reported VQoL scores stratified by participants’ demographic, familial and clinical characteristics are shown in Table 1. The median VQoL score was 55.58 (IQR = 12.06, min = 17.08, max = 100.00), and the median FV score was 50.90 (IQR = 12.57, min = 0.00, max = 100.00) in our sample. Additionally, the self-reported FV scores stratified by participants’ demographic, familial and clinical characteristics are shown in S1 Table.

**Table 1:** Self-reported vision-related quality of life scores stratified by sociodemographic, family, and clinical characteristics.

VQoL Score	*n*	Median	IQR	Minimum	Maximum	*p*-value
**All participants** ^**a**^	151	55.58	12.06	17.08	100	
**Gender**						
Male	84	55.58	9.445	17.08	100	.35^b^
Female	67	55.53	14.345	34.96	100	
**Age**						
7	3	64.53	8.600	50.89	68.09	.27^c^
8	19	54.84	6.185	48.28	71.15	
9	19	58.41	11.905	46.28	82.11	
10	9	50.89	10.380	45.59	64.53	
11	13	59.17	12.120	37.68	100	
12	17	59.17	5.960	40.35	75.30	
13	8	52.84	7.068	41.30	78.52	
14	19	57.86	8.870	34.96	66.82	
15	15	49.65	17.110	38.70	78.52	
16	13	53.48	8.640	17.08	69.69	
17	14	55.58	16.403	34.96	100	
18	2	54.42	5.980	48.44	60.40	
**Child’s Ethnicity**						
White UK	90	55.53	12.588	38.70	100	.47^d^
White other	7	48.94	7.365	40.35	59.97	
Black	11	57.08	15.845	34.96	100	
Mixed	6	57.93	11.193	48.94	75.30	
Asian	26	54.17	8.340	17.08	82.11	
Arab and other	3	57.99	8.425	52.84	69.69	
Missing	8					
**Socio-economic Status (IMD Quintile Rank)**						
1: most deprived area	37	54.16	9.930	17.08	100	.04^c^
2	26	55.56	11.840	34.96	78.52	
3	24	55.20	12.735	38.70	69.69	
4	27	59.52	13.630	44.31	100	
5: least deprived area	31	58.41	9.385	39.37	78.52	
Missing	6					
**Severity of Visual Impairment**						
VI (logMAR ≤ 1.00)^e^	120	55.90	11.095	34.96	100	.50^b^
SVI/Blind (logMAR ≥ 1.02)	31	54.84	11.915	17.08	100	
**Timing of Onset**						
Early (≤ 2 years after birth)	124	55.56	12.595	17.08	100	.61^b^
Late (> 2 years after birth)	27	55.58	10.125	34.96	75.30	
**Rate of Deterioration**						
Stable	108	55.20	12.463	34.96	100	.41^b^
Progressive	43	57.86	7.630	17.08	100	
**Presence of Additional Health Conditions or Impairments Affecting Development**						
No	110	55.90	12.580	34.96	100	.01^b^
Yes^**f**^	26	51.53	10.813	17.08	75.30	
Missing	15					
**Siblings**						
Yes	119	55.58	11.600	34.96	100	.16^b^
No	16	50.89	15.850	38.70	78.52	
Missing	16					
**Number of Siblings**						
One	43	55.58	13.670	34.96	82.11	.83^c^
Two	43	59.17	9.7650	40.35	78.52	
Three	10	52.19	7.328	34.96	65.62	
Four or more	10	56.33	8.705	41.30	69.69	
Missing	29	No siblings	16			
**Participant’s Birth Order**						
Youngest child	41	57.99	9.840	44.31	82.11	.28^d^
Oldest child	37	55.53	14.880	34.96	78.52	
Middle or twin	28	55.58	9.540	34.96	69.69	
Missing	29	No siblings	16			
**Family Structure**						
One-carer	18	56.72	18.228	34.96	75.30	.96^b^
Two-carers	118	55.53	10.455	17.08	100	
Missing	15					
**Type of School**						
Mainstream school	125	55.58	12.430	34.96	100.00	.01^b^
Other school types^g^	9	49.65	15.180	17.08	64.53	
Missing	17					
**Housing Tenure**						
Owned by the family	102	57.40	11.930	34.96	100	.09^d^
Rental	38	54.84	11.205	17.08	100	
Other (e.g., council house)	3	52.84	1.950	50.26	54.16	
Missing	8					
**Cars Owned**						
None	15	57.08	9.300	34.96	73.03	.08^c^
One	63	54.84	11.960	17.08	100	
Two or more	67	57.86	11.955	43.72	100	
Missing	6					
**Carer’s Age Group (years)**						
21 to 30	3	49.59	13.425	37.68	64.53	.28^c^
31 to 40	48	54.84	7.605	34.96	73.32	
41 to 50	76	55.58	13.885	17.08	100	
51 or more	18	58.20	7.093	44.31	78.52	
Missing	6					
**Carer’s Ethnicity**						
White UK	88	57.93	12.413	38.70	100	.34^d^
White other	13	52.81	8.920	40.35	71.15	
Black	12	57.08	13.113	34.96	100	
Mixed	3	49.65	3.640	48.94	56.22	
Asian	25	54.17	8.460	17.08	82.11	
Arab and other	5	52.84	7.730	46.07	69.69	
Missing	5					
**Carer’s Education and Qualification**						
No education, qualification	22	53.16	7.603	39.37	63.52	.02^c^
GCSE, four 0 levels	5	61.66	28.870	45.49	78.52	
A level, City Guilds, NVQ	46	55.56	13.715	34.96	73.32	
Professional qualification, Degree	65	57.99	12.220	37.68	100	
Missing	13					
**Carer’s Employment Status**						
Unemployed^h^	36	54.17	11.775	34.96	78.52	.20^b^
Employed	95	56.22	12.120	17.08	100	
Missing	20					
**Carer’s Employment by Required Skill Level**						
Level 4: the highest skill level	43	57.86	13.460	38.70	100	.59^c^
Level 3	18	55.90	8.830	34.96	82.11	
Level 2	26	57.11	12.743	17.08	78.52	
Level 1: the lowest skill level	4	52.83	2.533	45.49	55.53	
Missing	20	Unemployed	36	Undefined^i^	4	
**Carers’ Visual Impairment Status**						
One or both carers have VI	26	56.26	11.918	34.96	73.03	.88^b^
No VI	107	55.53	12.330	34.96	100	
Missing	18					
**Carers’ Medical Status**						
Has a chronic health condition	10	53.50	13.175	41.30	82.11	.79^b^
None	101	55.58	12.010	34.96	100	
Missing	40					
**Siblings’ Visual Impairment Status**						
With VI	36	55.90	13.428	34.96	82.11	.88^b^
Without VI	83	55.58	9.600	17.08	100	
Missing	16	No siblings	16			
**Siblings’ Medical Status**						
Has a chronic health condition	13	51.54	6.640	39.37	75.30	.16^b^
None	89	57.86	12.430	17.08	100	
Missing	33	No siblings	16			

GCSE, General Certificate of Secondary Education; IMD, Index of Multiple Deprivation; IQR, interquartile range; LogMAR, logarithm of minimum angle of resolution; NVQ, National Vocational Qualification; SVI/BL, severe visual impairment, or blindness; VI, visual impairment; VQoL, vision-related quality of life.

^a^ One participant with missing ≥20% of data on VQoL_CYP was excluded. The participant with missing ≥20% data on the FVQ_CYP instrument was only excluded in the analysis of FVQ_CYP.

^b^ Independent 2-samples Wilcoxon-Mann-Whitney *U* test.

^c^ Spearman’s rank correlation.

^d^ Independent samples Kruskal-Wallis test.

^e^ Three participants with visual acuity LogMAR 0.07–0.46 in the better eye or additional visual defects that classified them as visually impaired by WHO criteria were included.

^f^ This category CYP-VI who had at least one of the following pre-defined conditions: movement disorder, communication disorder, language disorder, behaviour disorder, developmental delay, epilepsy or seizure disorder, hearing impairment, eating disorder, or any other relevant health condition affecting their development.

^g^ This category included specialist schools (for students with visual impairment or medical conditions), home schooling, and other school types (e.g., community school).

^h^ Unemployed due to disability, being a student or stay-at-home carer.

^i^ Job position undefined as the carer reported only to be self-employed.

VQoL and FV scores were significantly negatively associated (*r*_Spearman_ = –0.52, *p <* .001), showing CYP-VI who reported better VQoL also often reported less FV difficulties. CYP-VI with severe VI or blindness reported significantly more FV difficulty (*r*_Spearman_ = 0.30, *p <* .001) but not significantly worse VQoL (*r*_Spearman_ = –0.06, *p =* .50) than CYP with moderate VI (as reported in the validation study) [40, 42].

The presence of additional diagnoses affecting a child’s development was associated with both FV scores (*p =* .02, *d*_Cohen_ = 0.41) and VQoL scores (*p =* .01, *d*_Cohen_ = 0.46), indicating CYP-VI who had one or more additional diagnoses (*n* = 26) reported worse VQoL and more FV problems than those without an additional diagnosis (*n* = 110).

Furthermore, VQoL scores significantly correlated with the IMD quintile rank (*r*_Spearman_ = 0.17, *p =* .04) and carer’s education level (*r*_Spearman_ = 0.20, *p =* .02), demonstrating CYP-VI with less educated carers and living in areas with lower IMD quintile rank usually reported worse VQoL. Carer’s education level was also significantly associated with FV scores (*r*_Spearman_ = –0.18, *p =* .04), indicating CYP-VI with less educated parents also reported more functional vision difficulties.

CYP-VI attending mainstream school (*n* = 125) reported significantly better VQoL (*p =* .01, *d*_Cohen_ = 0.44) and FV (*p =* .02, *d*_Cohen_ = 0.42) compared to CYP-VI enrolled in other types of school (*n* = 9).

No other associations or comparisons between the VQoL or FV scores and the selected factors were statistically significant.

Table 2 shows quantile regression models for each variable used in the analyses. VQoL scores had a significant relationship with functional vision scores, presence of additional health conditions or impairments, IMD, and parental education level, but not with the type of school a child was enrolled in. As Table 3 shows, our final quantile regression model included two variables–functional vision and the presence of additional health condition or impairment. Both had a significant negative relationship with the outcome measure. The predicted median value of VQoL score would decrease by 0.362 point with every 1-point increase in FV score and by 3.904 points in the presence of additional health conditions or impairments.

**Table 2:** Quantile regression models for vision-related quality of life of children and young people with visual impairment.

Variable	Coefficient	Standard Error	95% Confidence Intervals (Cis)	*p-*value
**Functional Vision**				
Intercept	76.387	4.609	[73.16; 86.57]	< .001
Functional Vision	–0.414	0.092	[–0.59; –0.34]	< .001
**Presence of Additional Health Conditions or Impairments**				
Intercept	55.580	1.299	[53.71; 60.26]	< .001
Presence of Additional Health Conditions or Impairments	–4.040	2.002	[–12.08; –1.37]	.046
**Type of School**				
Intercept	55.580	1.256	[53.66; 60.30]	< .001
Type of School	–5.930	4.371	[–15.15; –2.48]	.18
**Index of Multiple Deprivation (IMD) Quantile Rank**				
Intercept	53.098	1.622	[49.57; 54.36]	< .001
IMD Quantile Rank	1.063	0.484	[0.52; 1.81]	.03
**Parental Education Level**				
Intercept	52.840	1.632	[49.64; 55.63]	< .001
Parental Education Level	1.690	0.688	[0.70; 3.69]	.02

**Table 3:** Final quantile regression model for vision-related quality of life of children and young people with visual impairment.

Variable	Coefficient	Standard Error	95% Confidence Intervals (Cis)	*p-*value
Intercept	75.110	4.658	[69.95; 85.73]	< .001
Functional Vision	–0.362	0.092	[–0.58; –0.27]	< .001
Presence of additional health conditions or impairments	–3.904	1.782	[–5.60; –0.68]	.03

## Discussion

From this cross-sectional study of a representative sample of CYP-VI, we report to our knowledge for the first time sociodemographic and broader family factors that influence self-reported VQoL.

Identifying factors that shape the life experience of CYP-VI is an integral part of identifying those at greatest risk of low VQoL individuals (i.e., greatest need) and prioritising potential targets for interventions to improve VQoL outcomes of these patients, thereby improving personalised care. The value of PROMs lies in their ability to capture the broader impact of living with a visual disability from the perspective of CYP-VI and in identifying the impact of age- and condition-specific challenges they experience.

Limitations of this study relate to its use of data previously collected within a broader research programme, which means there was limited power for statistical analysis of some variables of interest, such as subsamples of CYP-VI with additional diagnosis or those not attending mainstream schools. As the data were collected via a postal survey, there is no knowing if carers influenced self-reporting by CYP-VI. Finally, the cross-sectional design means we cannot infer causality, which could be addressed in future longitudinal studies. The strengths of this study are the assessment of a large number of pre-specified potential associated variables with VQoL, including individual, family, and broader environmental factors and the use of robust validated PROMs to capture a secure measure of the impact of VI in childhood.

CYP-VI with better self-assessed VQoL also reported better self-perceived functional vision [39]. However, as previously reported [40], the severity of visual impairment was not associated with VQoL of CYP-VI. This contrasts with the relationship between the severity of VI and FV scores [42]. These results support the notion of a disability paradox, where individuals can report good QoL despite their disability or poor functioning [25–27, 40]. Equally, it shows VQoL and FV are two separate constructs, although CYP-VI who self-perceive having poor FV seem to be at risk of poor self-perceived VQoL. This underlines the strong need for clinicians to avoid presupposing the VQoL of their patients based on the severity of functional impairment.

In accordance with previous findings [36], we found CYP-VI with additional chronic health conditions or impairments affecting development, self-reported worse VQoL and greater FV difficulties. This demonstrates the impact of multi-morbidity and identifies a sub-group at higher risk of adverse VQoL.

Similar to prior reports [37, 38], our results showed key sociodemographic characteristics, such as age, gender, and ethnicity, were not significantly related to VQoL and FV of CYP-VI. However, the carer’s education level and socio-economic status were significantly associated with VQoL, with CYP-VI living in more socio-economically deprived areas and with less educated carers reporting worse VQoL. These findings are in line with a systematic review highlighting the carer’s education level and household income as the two most frequently reported socio-economic determinants of QoL of children and young people with different chronic health conditions [28]. This suggests that inequalities in VQoL are not pre-destined through demographic characteristics and identifies the scope for reducing inequalities through societal interventions.

The finding that CYP-VI in mainstream schools (with or without a specialist VI unit) experienced better VQoL than CYP-VI who were home-schooled or enrolled in a specialist and other types of school is interesting and concords with previous research showing that CYP-VI in specialist schools had worse daily living skills, social skills [16], and lower level of adjustment to their disability [17] compared to CYP-VI in mainstream schools. Of course, several factors determine whether a child living with VI attends a mainstream school. It is likely that, on average, CYP-VI attending other types of school might have greater health needs and functioning difficulties that impact activities of daily living, which require more support due to having complex chronic health conditions, and all this could adversely impact their VQoL. It is plausible that CYP-VI in mainstream schools, assuming they receive adequate specific VI learning support, have greater opportunities to socialise broadly and to become more independent, self-confident, and resilient.

Even though it may not be possible to directly target factors such as, for example, living in more deprived areas or multi-morbidity, it is important to be mindful of these potential risks when designing interventions and allocating resources. Our findings show that interventions aimed at improving quality of life of CYP-VI should consider targeting resources at families of CYP-VI with more complex healthcare and clinical needs and those from lower socioeconomic groups. In the light of the recent COVID-19 pandemic, the cost-of-living crisis, and the higher incidence of VI in families living in the most deprived areas [4], further research into the potential worsening of health inequalities in paediatric ophthalmology is crucial.

From a clinical perspective, ensuring multidisciplinary support and providing a personalised approach to the specific healthcare needs of patients at higher risk may modify or eliminate the potential negative impact of the risk factors (e.g., having an additional chronic health condition). This requires further research to examine if such interventions and considerations would be helpful. Nevertheless, our findings demonstrate markers that clinicians can use to identify those amongst their patients who are likely to be at greatest risk of lower quality of life and, therefore, potentially in greatest need so that they are able to direct these patients to existing resources and services.

Overall, as the impact of childhood VI may vary significantly across CYP-VI, there is a need for a more personalised approach to their care. The VQoL_CYP and FVQ_CYP are validated tools that can be easily utilised in routine clinical practice to monitor these patients’ outcomes [44] by assessing life experience from child’s perspective and providing the clinical team with information on the impact VI and any treatment has on their patient.

## Conclusions

In conclusion, our study identifies that certain CYP-VI are at greater risk of experiencing poor vision-related quality of life. Socio-economic factors are at least as important as the clinical factors that healthcare professionals usually consider when thinking about which patients may need additional attention or support. Multidisciplinary teams have an important role in meeting the needs of affected children and their families to help them achieve better VQoL, especially in providing personalised care. School presents an important context for CYP-VI, and further research examining the impact of school setting on VQoL is warranted. Research to improve VQoL should consider clinical (e.g., multi-morbidity) as well as broader family and socio-economic determinants as potential targets for intervention.

## Supporting information

S1 File Coding of the family and sociodemographic characteristics. This supplemental material has been provided to give readers detailed information about the coding of the answers that parents/carers provided in the family background questionnaire.
(DOCX)

S1 Table Self-reported functional vision scores stratified by sociodemographic, family, and clinical characteristics. (DOCX)
