# Supplementary material for: Individual, family, and environmental determinants of vision-related quality of life of children and young people with visual impairment
Source: PLoS One. 2023 Nov 16;18(11):e0294532. doi: 10.1371/journal.pone.0294532 (PMC10653485; doi:10.1371/journal.pone.0294532)
Supplement: S1 File — This supplemental material has been provided to give readers detailed information about the coding of the answers that parents/carers provided in the family background questionnaire. (DOCX) [file pone.0294532.s001.docx]

**S1 File**

**Coding of the family and sociodemographic characteristics**

This supplemental material has been provided to give readers a detailed information about the coding of the answers that parents/carers provided in the family background questionnaire.

*Presence of additional health, sensory, and/or behavioural diagnoses* was categorised as ‘Yes’ if a carer reported their child had at least one of the nine pre-defined conditions (“movement disorder”, “communication disorder”, “language disorder”, “behaviour disorder”, “developmental delay”, “epilepsy or seizure disorder”, “hearing impairment”, “eating disorder”, and “other, please specify”). The category ‘No’ included children with no reported additional diagnoses that affected development.

*Participants’ ethnicity* and *carer's ethnicity* were categorised using Office for National Statistics (ONS) guidelines [1].

*Participant’s birth order* was coded based on age of siblings reported by their carer.

*Carer’s education level* was categorised using the ONS guidelines [2]. The lowest category of education comprised ‘No qualifications or training’, followed by ‘General Certificate of Secondary Education (GCSE)’ and ‘Four 0 levels’. The third category combined ‘A level’, ‘City Guilds Certificate’ and ‘National Vocational Qualification’. The highest level comprised ‘Professional qualification’ and ‘Degree’.

*Carer’s occupational level* was first grouped using carer’s job position and ONS Standard Occupational Classification Hierarchy [3], and then categorised based on the required skill level for the respondent carer's job position following ONS guidelines [4].

*Index of Multiple Deprivation quintile rank* (IMD) [5] was assigned to each participant based on their UK postcode. This metric takes account of income, employment, education, health, crime, barriers to housing and services, and living environment, as the UK’s standard multicomponent metric of relative deprivation for small areas.

*Housing tenure* was categorised as ‘Owned by the family’, or ‘Rented’ (privately, from a housing association, or local authority) or ‘Other’.

*Type of school* was categorised as ‘Mainstream’ which included mainstream school with specialist visual impairment unit. ‘Other school types’ included any type of specialist schools, home schooling, and all other school types as specified by carers.

**References**

1. Office for National Statistics. Ethnicity and National Identity in England and Wales: 2011. Published 2012. Accessed October 25, 2020. https://www.ons.gov.uk/peoplepopulationandcommunity/culturalidentity/ethnicity/articles/ethnicityandnationalidentityinenglandandwales/2012-12-11#measuring-ethnicity

2. 2011 Census - Office for National Statistics. Accessed June 29, 2022. https://www.ons.gov.uk/employmentandlabourmarket/peopleinwork/employmentandemployeetypes/bulletins/keystatisticsandquickstatisticsforlocalauthoritiesintheunitedkingdom/2013-12-04

3. Office for National Statistics. ONS Standard Occupational Classification (SOC) Hierarchy. Office for National Statistics. Accessed December 6, 2020. https://onsdigital.github.io/dp-classification-tools/standard-occupational-classification/ONS_SOC_hierarchy_view.html

4. Office for National Statistics. SOC 2020 Volume 1: structure and descriptions of unit groups. Accessed October 25, 2020. https://www.ons.gov.uk/methodology/classificationsandstandards/standardoccupationalclassificationsoc/soc2020/soc2020volume1structureanddescriptionsofunitgroups

5. Ministry of Housing, Communities &, Local Government. English indices of deprivation 2015. GOV.UK. Published 2015. Accessed May 22, 2020. https://www.gov.uk/government/statistics/english-indices-of-deprivation-2015
