# Supplementary material for: Individual, family, and environmental determinants of vision-related quality of life of children and young people with visual impairment
Source: PLoS One. 2023 Nov 16;18(11):e0294532. doi: 10.1371/journal.pone.0294532 (PMC10653485; doi:10.1371/journal.pone.0294532)
Supplement: S1 Table — (DOCX) [file pone.0294532.s002.docx]

**S2 Table**

**Table. Self-Reported Functional Vision Scores Stratified by Sociodemographic, Family, And Clinical Characteristics.**

| **Functional Vision Score** | ***n*** | | | | **Median** | | **IQR** | **Minimum** | | **Maximum** | | ***p-*value** |
| --- | --- | --- | --- | --- | --- | --- | --- | --- | --- | --- | --- | --- |
| **All participants** | 150 | | | | 50.90 | | 12.163 | 0 | | 100 | |  |
| *Missing* | 1 | | | |  | |  |  | |  | |  |
| **Gender** | | | | | | | | | | | | |
| Male | 84 | | | | 50.67 | | 9.993 | 0 | | 100 | | .32^a^ |
| Female | 66 | | | | 51.34 | | 11.838 | 0 | | 77.04 | |  |
| **Age** | | | | | | | | | | | | |
| 7 | 3 | | | | 48.11 | | 2.835 | 44.49 | | 50.16 | | .53^b^ |
| 8 | 18 | | | | 50.68 | | 6.188 | 29.57 | | 67.41 | |  |
| 9 | 19 | | | | 52.22 | | 12.555 | 29.57 | | 63.45 | |  |
| 10 | 9 | | | | 53.27 | | 9.660 | 41.77 | | 61.39 | |  |
| 11 | 13 | | | | 50.67 | | 12.020 | 0 | | 72.59 | |  |
| 12 | 17 | | | | 51.19 | | 8.860 | 24.55 | | 75.39 | |  |
| 13 | 8 | | | | 53.52 | | 11.568 | 39.45 | | 72.24 | |  |
| 14 | 19 | | | | 47.95 | | 13.030 | 31.65 | | 100 | |  |
| 15 | 15 | | | | 55.05 | | 19.580 | 25.82 | | 66.93 | |  |
| 16 | 13 | | | | 51.88 | | 12.390 | 33.60 | | 100 | |  |
| 17 | 14 | | | | 50.71 | | 7.618 | 22.18 | | 59.61 | |  |
| 18 | 2 | | | | 48.43 | | 5.825 | 42.60 | | 54.25 | |  |
| **Child’s Ethnicity** | | | | | | | | | | | | |
| White UK | 90 | | | | 50.69 | | 9.298 | 22.18 | | 77.04 | | .32^c^ |
| White other | 7 | | | | 52.75 | | 13.205 | 38.49 | | 75.39 | |  |
| Black | 10 | | | | 51.07 | | 30.088 | 0 | | 58.33 | |  |
| Mixed | 6 | | | | 51.86 | | 7.833 | 29.57 | | 61.39 | |  |
| Asian | 26 | | | | 54.61 | | 14.043 | 29.57 | | 100 | |  |
| Arab and other | 3 | | | | 42.33 | | 4.450 | 39.44 | | 48.34 | |  |
| *Missing* | 8 | | | |  | |  |  | |  | |  |
| **Socio-economic Status (IMD Quintile Rank)** | | | | | | | | | | | | |
| 1: most deprived area | 36 | | | | 53.27 | | 13.408 | 0 | | 100 | | .20^b^ |
| 2 | 26 | | | | 50.16 | | 11.338 | 25.82 | | 100 | |  |
| 3 | 24 | | | | 52.32 | | 7.003 | 34.79 | | 77.04 | |  |
| 4 | 27 | | | | 48.62 | | 7.750 | 0 | | 64.26 | |  |
| 5: least deprived area | 31 | | | | 52.22 | | 9.750 | 22.18 | | 65.82 | |  |
| *Missing* | 6 | | | |  | |  |  | |  | |  |
| **Severity of Visual Impairment** | | | | | | | | | | | | |
| VI (logMAR ≤ 1.00) | 119 | | | | 49.92 | | 11.610 | 0 | | 77.04 | | <.001^a^ |
| SVI/Blind (logMAR ≥ 1.02) | 31 | | | | 57.91 | | 15.345 | 38.00 | | 100 | |  |
| **Timing of Onset** | | | | | | | | | | | | |
| Early (≤ 2 years after birth)^d^ | 123 | | | | 50.16 | | 11.480 | 0 | | 100 | | .05^a^ |
| Late (> 2 years after birth) | 27 | | | | 52.75 | | 12.840 | 41.20 | | 100 | |  |
| **Rate of Deterioration** | | | | | | | | | | | | |
| Stable | 108 | | | | 50.42 | | 10.613 | 0 | | 74.88 | | .23^a^ |
| Progressive | 42 | | | | 51.97 | | 14.070 | 0 | | 100 | |  |
| **Presence of Additional Diagnoses Affecting Development** | | | | | | | | | | | | |
| No | 109 | | | | 50.16 | | 11.490 | 0 | | 77.04 | | .02^a^ |
| Yes^e^ | 26 | | | | 54.89 | | 9.313 | 24.55 | | 100 | |  |
| *Missing* | 15 | | | |  | |  |  | |  | |  |
| **Siblings** | | | | | | | | | | | | |
| Yes | 118 | | | | 51.14 | | 10.550 | 0 | | 100 | | .46^a^ |
| No | 16 | | | | 54.84 | | 12.675 | 31.65 | | 66.93 | |  |
| *Missing* | 16 | | | |  | |  |  | |  | |  |
| **Number of Siblings** | | | | | | | | | | | | |
| One | 43 | | 51.88 | | | 9.225 | | 29.57 | 72.59 | | .98^b^ | |
| Two | 42 | | 50.68 | | | 12.623 | | 22.18 | 75.39 | |  |  |
| Three | 10 | | 54.08 | | | 7.223 | | 29.57 | 100 | |  |  |
| Four or more | 10 | | 50.51 | | | 7.835 | | 34.78 | 77.04 | |  |  |
| *Missing* | 29 | | *No siblings* | | | 16 | |  |  | |  | |
| **Participant’s Birth Order** | | | | | | | | | | | | |
| Youngest child | 41 | | 50.70 | | | 9.010 | | 29.57 | 72.59 | | .28^c^ | |
| Oldest child | 36 | | 51.34 | | | 12.138 | | 22.18 | 75.39 | |  |  |
| Middle or twins | 28 | | 54.08 | | | 10.478 | | 38.21 | 100 | |  |  |
| *Missing* | 29 | | *No siblings* | | | 16 | |  |  | |  | |
| **Family Structure** | | | | | | | | | | | | |
| One-carer | 18 | | 53.85 | | | 5.368 | | 22.18 | 77.04 | | .35^a^ | |
| Two-carers | 117 | | 50.67 | | | 12.740 | | 0 | 100 | |  |  |
| *Missing* | 15 | |  | | |  | |  |  | |  | |
| **Type of School** | | | | | | | | | | | | |
| Mainstream school | 124 | | 50.69 | | | 10.200 | | 0 | 100 | | .02^a^ | |
| Other school types^f^ | 9 | | 58.33 | | | 6.440 | | 45.94 | 100 | |  |  |
| *Missing* | 17 | |  | | |  | |  |  | |  | |
| **Housing Tenure** | | | | | | | | | | | | |
| Owned by the family | 102 | | 50.69 | | | 9.790 | | 22.18 | 100 | | .99^c^ | |
| Rental | 37 | | 49.92 | | | 13.200 | | 0 | 100 | |  |  |
| Other (e.g., council house) | 3 | | 51.09 | | | 10.750 | | 39.44 | 60.94 | |  |  |
| *Missing* | 8 | |  | | |  | |  |  | |  | |
| **Cars Owned** | | | | | | | | | | | | |
| None | | 15 | | | 45.13 | | 14.510 | 0 | | 61.39 | | .62^b^ |
| One | | 62 | | | 51.45 | | 12.238 | 20.88 | | 100 | |  |
| Two or more | | 67 | | | 50.16 | | 10.215 | 0 | | 72.59 | |  |
| *Missing* | | 6 | | |  | |  |  | |  | |  |
| **Carer’s Age Group (years)** | | | | | | | | | | | | |
| 21 to 30 | | 2 | | | 58.69 | | 2.705 | 55.98 | | 61.39 | | .79^b^ |
| 31 to 40 | | 48 | | | 50.16 | | 11.998 | 0 | | 100 | |  |
| 41 to 50 | | 76 | | | 51.14 | | 11.388 | 0 | | 100 | |  |
| 51 or more | | 18 | | | 46.77 | | 18.230 | 25.82 | | 77.04 | |  |
| *Missing* | | 6 | | |  | |  |  | |  | |  |
| **Carer’s Ethnicity** | | | | | | | | | | | | |
| White UK | | 88 | | | 50.42 | | 9.400 | 22.18 | | 77.04 | | .33^c^ |
| White other | | 13 | | | 47.60 | | 7.620 | 38.49 | | 75.39 | |  |
| Black | | 11 | | | 47.09 | | 28.880 | 0 | | 58.33 | |  |
| Mixed | | 3 | | | 54.34 | | 4.585 | 52.22 | | 61.39 | |  |
| Asian | | 25 | | | 54.88 | | 15.200 | 29.57 | | 100 | |  |
| Arab and other | | 5 | | | 48.34 | | 8.760 | 39.44 | | 66.93 | |  |
| *Missing* | | 5 | | |  | |  |  | |  | |  |
| **Carer’s Education and Qualification** | | | | | | | | | | | | |
| No education, qualification | | 21 | | | 52.75 | | 13.620 | 29.57 | | 74.88 | | .04^b^ |
| GCSE, four 0 levels | | 5 | | | 47.95 | | 6.700 | 39.45 | | 65.82 | |  |
| A level, City Guilds, NVQ | | 46 | | | 53.24 | | 10.113 | 20.88 | | 100 | |  |
| Professional qualification, Degree | | 65 | | | 48.62 | | 12.550 | 0 | | 75.39 | |  |
| *Missing* | | 13 | | |  | |  |  | |  | |  |
| **Carer’s Employment Status** | | | | | | | | | | | | |
| Unemployed^g^ | | 35 | | 53.27 | | | 13.495 | 0 | | 100 | | .08^a^ |
| Employed | | 95 | | 50.16 | | | 11.740 | 0 | | 100 | |  |
| *Missing* | | 20 | |  | | |  |  | |  | |  |
| **Carer’s Employment by Required Skill Level** | | | | | | | | | | | | |
| Level 4: the highest skill level | | 42 | | 50.16 | | | 10.215 | 0 | | 65.82 | | .80^b^ |
| Level 3 | | 18 | | 47.86 | | | 13.875 | 24.55 | | 64.26 | |  |
| Level 2 | | 25 | | 50.93 | | | 8.420 | 22.18 | | 100 | |  |
| Level 1: the lowest skill level | | 4 | | 53.10 | | | 26.538 | 39.44 | | 69.27 | |  |
| *Missing* | | 22 | | *Unemployed* | | | 35 | *Undefined*^h^ | | 4 | |  |
| **Carers’ Visual Impairment Status** | | | | | | | | | | | | |
| One or both carers have VI | | 25 | | 52.22 | | | 8.700 | 0 | | 75.39 | | .72^a^ |
| Without VI | | 107 | | 51.19 | | | 12.340 | 0 | | 100 | |  |
| *Missing* | | 18 | |  | | |  |  | |  | |  |
| **Carers’ Medical Status** | | | | | | | | | | | | |
| Has a chronic health condition | | 10 | | 51.71 | | | 11.600 | 29.57 | | 62.06 | | .59^a^ |
| None | | 101 | | 50.70 | | | 11.140 | 0 | | 100 | |  |
| *Missing* | | 39 | |  | | |  |  | |  | |  |
| **Siblings’ Visual Impairment Status** | | | | | | | | | | | | |
| With VI | | 35 | | 50.16 | | | 12.560 | 29.57 | | 100 | | .18^a^ |
| Without VI | | 83 | | 51.19 | | | 11.820 | 0 | | 100 | |  |
| *Missing* | | 16 | | *No siblings* | | | 16 |  | |  | |  |
| **Siblings’ Medical Status** | | | | | | | | | | | | |
| Has a chronic health condition | | 13 | | 54.34 | | | 10.200 | 38.49 | | 77.04 | | .06^a^ |
| None | | 89 | | 50.67 | | | 9.490 | 0 | | 100 | |  |
| *Missing* | | 32 | | *No siblings* | | | 16 |  | |  | |  |
| GCSE, General Certificate of Secondary Education; IMD, Index of Multiple Deprivation; IQR, interquartile range; LogMAR, logarithm of minimum angle of resolution; NVQ, National Vocational Qualification; SVI/BL, severe visual impairment, or blindness; VI, visual impairment.  ^a^ Independent 2-samples Wilcoxon-Mann-Whitney *U* test.  ^b^ Spearman’s rank correlation.  ^c^ Independent samples Kruskal-Wallis test.  ^d^ Three participants with visual acuity LogMAR 0.07-0.46 in the better eye, and additional visual defects that classified them as visually impaired by WHO criteria were included.  ^e^ This category CYP-VI who had at least one of the following pre-defined conditions: movement disorder, communication disorder, language disorder, behaviour disorder, developmental delay, epilepsy or seizure disorder, hearing impairment, eating disorder, or any other relevant health condition affecting their development.  ^f^ This category included specialist schools (for CYP with VI or medical conditions), home schooling, and other school types (e.g., community school).  ^g^ Job position undefined as the carer reported only to be self-employed.  ^h^ Unemployed due to disability, being a student or stay-at-home carer. | | | | | | | | | | | | |
